# Systemic Lupus Erythematosus and Dermatomyositis Overlap Syndrome: Diagnostic Challenges and Management Insights

**DOI:** 10.7759/cureus.78424

**Published:** 2025-02-03

**Authors:** Saif-Eddin Dabour, Asmaa Elbenni, Ahmed M Abdel-Megid, Walid Alorfali

**Affiliations:** 1 Internal Medicine, Medical University of Gdansk, Gdansk, POL; 2 Rheumatology, Rheumatology Center of New Jersey, Somerville, USA

**Keywords:** anti-mda5 dermatomyositis, class v lupus nephritis, ivig therapy, sle-myositis overlap syndrome, systemic lupus erythromatosus

## Abstract

We present the case of a 27-year-old female patient who was initially diagnosed with dermatomyositis and later found to have an overlap with systemic lupus erythematosus (SLE). Despite extensive treatment with immunosuppressive medication, the patient experienced multiple deteriorations and treatment-related complications with waxing and waning improvement. This case aims to underscore the challenges involved in diagnosing and managing overlap syndromes and highlights the importance of comprehensive diagnostic workup and multidisciplinary care.

## Introduction

Dermatomyositis (DM) is an autoimmune muscular inflammatory disease that usually presents with pathognomonic markers such as Gottron’s papules and a heliotrope rash and generalized ones such as elevated creatine kinase and proximal muscle weakness [1]. Systemic lupus erythematosus (SLE), on the other hand, is another autoimmune inflammatory condition that is systemic in nature and affects multiple organs and systems in the body, and has its own set of criteria for diagnosis [2]. This case is about a young female patient of African descent who developed an overlap syndrome of dermatomyositis and SLE. She was originally diagnosed and treated for DM for four years before presenting to our clinic. This case report should raise awareness of the young age of onset of such an overlap disorder and its atypical presentation.

## Case presentation

**Figure 1:**
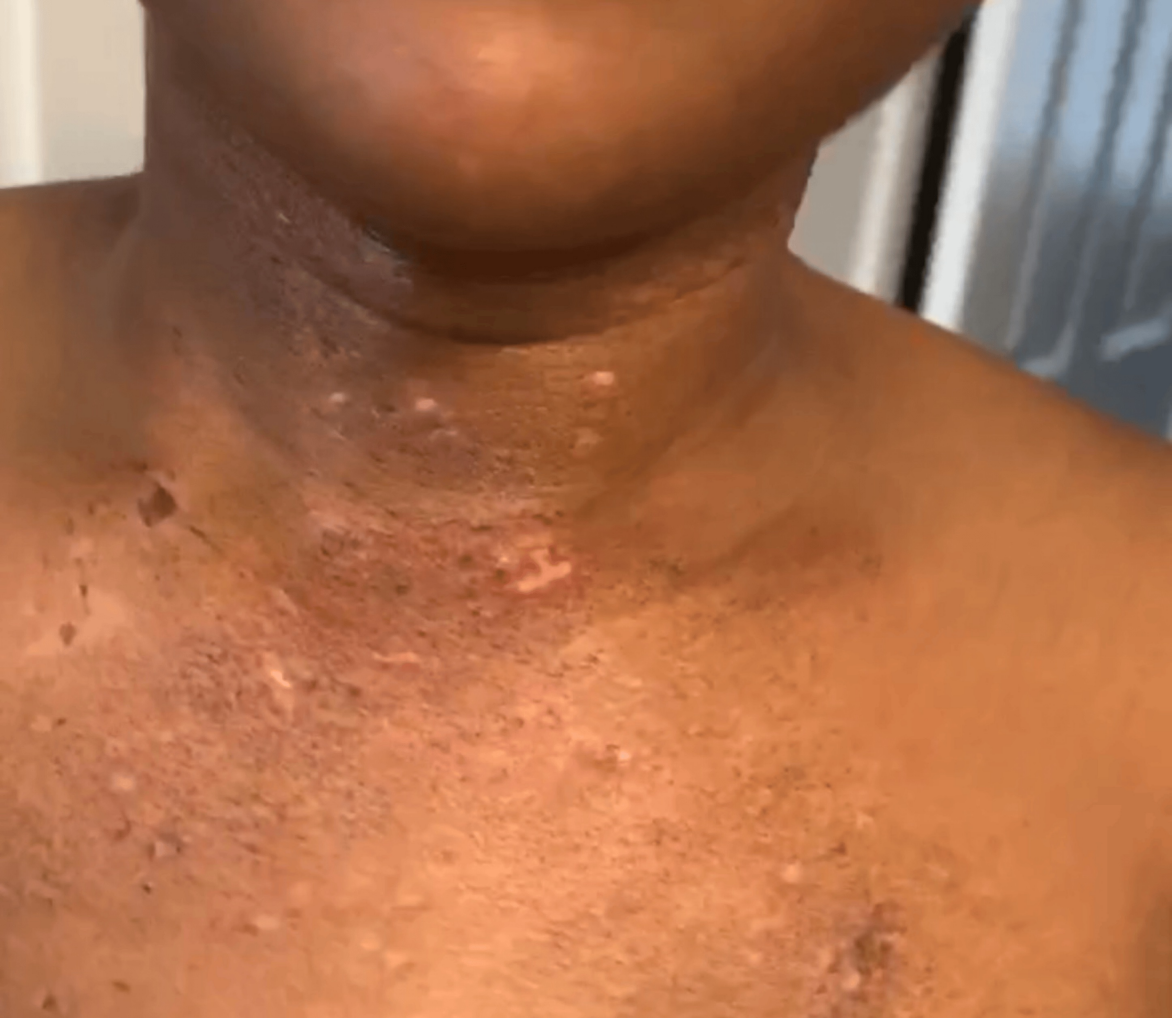
Erythema with subtle dyschromia on the anterior aspect of the neck and upper chest

**Figure 2:**
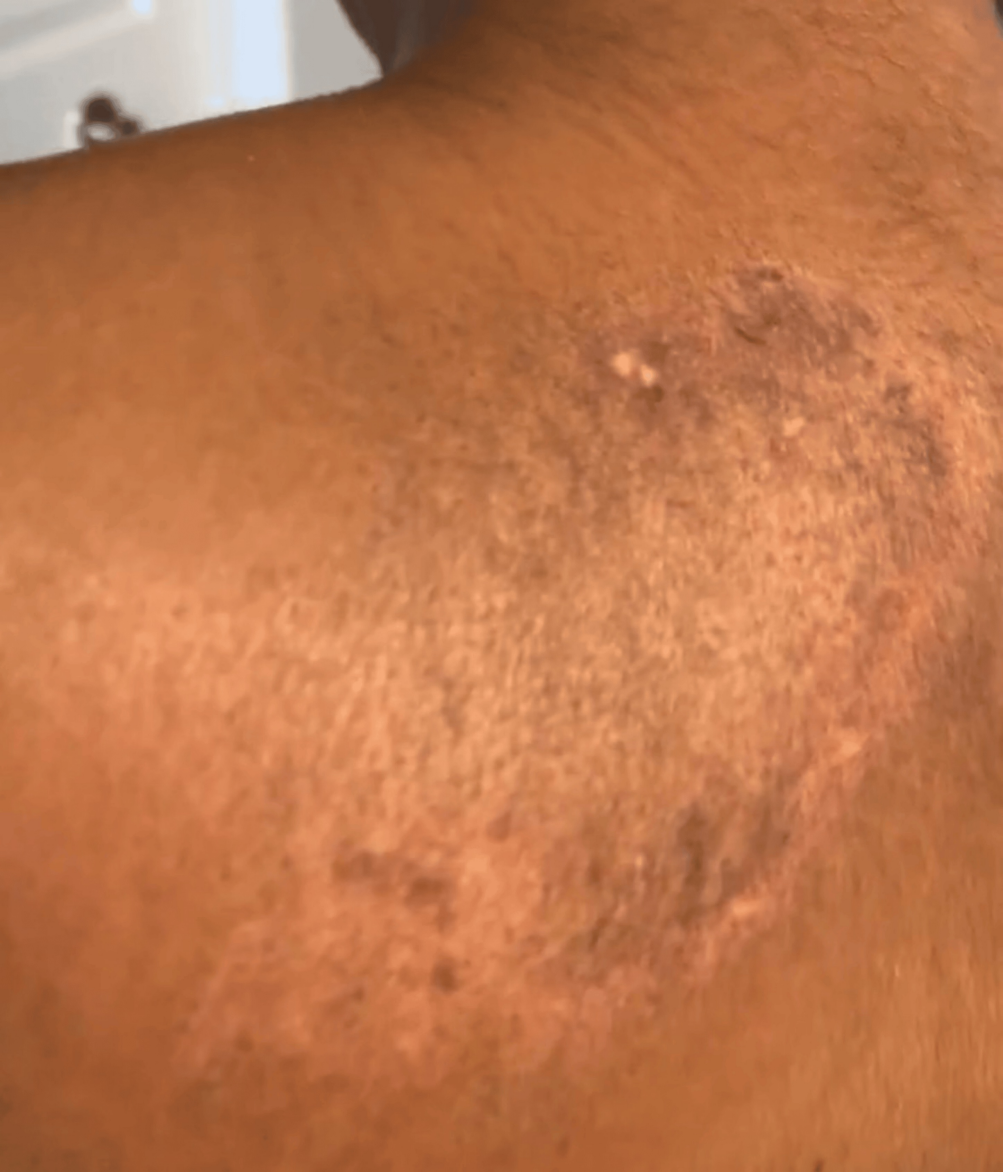
Dyschromia with subtle erythema on the left upper back

A 27-year-old female patient of African descent with a history of dermatomyositis and anxiety presented to our clinic in 2023 after moving states within the continental United States. She had been previously diagnosed with DM in late 2019; at first her facial puffiness as well as upper chest (Figure 1) and upper back (Figure 2) "rash" were thought to be an allergic reaction. She was seen by an allergist who prescribed treatment for dust mites. A few months later, she developed proximal muscle weakness and fatigue.

A muscle biopsy was performed and confirmed dermatomyositis; her rheumatologist then started treatment with 90 mg of prednisone daily. Multiple therapies were tried, including high-dose prednisone, steroid tapers, intravenous immunoglobulin (IVIG) infusions, methotrexate, azathioprine, mycophenolate mofetil, and hydroxychloroquine. Methotrexate was discontinued due to elevated liver function tests (LFTs), azathioprine due to diarrhea, and mycophenolate mofetil due to abdominal pain and weight loss. Hydroxychloroquine was discontinued due to a lack of efficacy. Her myositis-specific antibody panel was positive for anti-TIF 1 gamma (75; normal range <11), anti-OJ (14; normal range <11), and anti-MDA-5: (15; normal range <11). Anti-OJ and anti-MDA-5 were negative on repeat testing.

Her lab results at the time of referral showed an elevated creatine phosphokinase (CPK) (569 U/L; upper limit 192 U/L), anemia with hemoglobin (10.2 g/dL; normal range 11.2-15.7 g/dL), platelet count (762,000/mmc; normal range: 150,000-450,000/mmc), and aspartate aminotransferase (AST) (40 U/L; upper limit <32 U/L).

Prednisone and intravenous immunoglobulin (IVIG) therapy seemed to provide the most benefit to the patient, and her case stabilized. In 2022, she had a bout of COVID-19. Her situation regressed massively, and she had severe muscle weakness. She had extreme difficulty dressing herself. At this point, she had been on 40 mg of prednisone daily, which was complicated by bilateral femoral avascular necrosis without a fracture. Her rheumatologist decided to start rituximab infusions in early 2023.

She presented to our practice in May 2023 to transfer care. At the time of her arrival, her treatment consisted of 15 mg of prednisone orally daily, IVIG infusion twice monthly, and rituximab infusions. Comprehensive serologies in June 2023 are shown below (Table 1). AST was normal on repeat testing. Rheumatoid arthritis, SLE, systemic sclerosis, and vasculitis-specific antibodies, including anti-Jo-1, -MPO, -PR3, -cardiolipin, -centromere, -dsDNA, -CCP, and -histone, were negative.

**Table 1:** June 2023 serologies Patient's first visit to our clinic. Ant-Sm/RNP: anti-Smith/ribonucleoprotein antibody; ANA: antinuclear antibody; CPK: creatine phosphokinase

Particulars	Result	Reference
ANA titer	1:1280 nuclear, speckled	<1:80
Anti-Sm	>8.0	<1.0
Anti-Sm/RNP	>8.0	<1.0
CPK	447 U/L	29-143 U/L
C-reactive protein	11.6 mg/L	<8.0 mg/L
Creatinine	0.38 mg/dL	0.50-0.96 mg/dL
Hemoglobin	11.4 g/dL	11.7-15.5 g/dL
Platelet count	672,000/mmc	140,000-400,000/mmc
Protein/Creatinine ratio	2000 mg/g	24-184 mg/g
Urinalysis protein	2+	Negative

On a physical exam, there was active synovitis in the elbows, knees, and wrists bilaterally, and generalized muscle weakness. We also noted hypopigmented lesions on her axilla that did not seem related to the dermatomyositis. A skin biopsy performed to rule out psoriasis was highly suggestive of bullous systemic lupus erythematosus. She was started on a 20 mg prednisone taper and IVIG infusions before transitioning to rituximab. The patient was started on rituximab infusion IV 1 gram twice biweekly every six months. We were concerned about chronic steroid use to relieve the patient of her symptoms since she had a history of bilateral femoral head avascular necrosis. She was taking 10 mg of prednisone orally daily, but we reduced it to 5 mg. She was then started on repository corticotropin injection (RCI, Acthar® Gel). 

Four months later, the patient came back for a follow-up visit. She had improved greatly, but her disease was progressing. Her kidney function improved on the RCI; the protein/creatinine ratio improved from 2000 mg/g to 1068 mg/g. Her creatine kinase was 550 U/L (upper limit 143 U/L), and her platelets were 450,000/mmc (normal range 140,000-400,000/mmc). We reduced her RCI from twice weekly to once weekly due to adverse effects and referred the patient for a renal biopsy. The results of the biopsy confirmed our doubts and showed diffuse capillary loop immune complex deposits that were 3+ for IgG and 1+ for C3. The patient was then referred to Johns Hopkins Myositis Center for further evaluation and management. 

**Figure 3:**
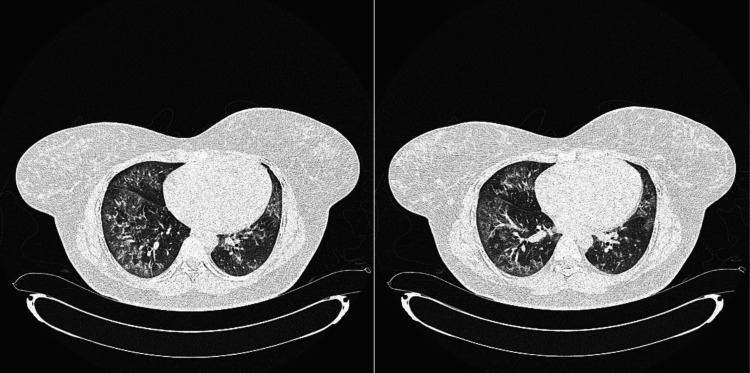
Non-contrast chest CT revealing diffuse ground glass opacities and bronchocentric patchy areas of consolidation

The pulmonologist suspected she had pulmonary hypertension; hence, she was referred to cardiology for an echocardiogram. Echocardiography was negative for pulmonary hypertension or sleep apnea. A non-contrast CT (computed tomography) performed in April 2024 revealed bilateral ground glass opacities at the bases with a dermatomyositis-associated interstitial lung disease (ILD) pattern (Figure 3). Pulmonary function tests (PFTs) revealed moderate to severe restrictive lung disease and reduced diffusion capacity. Her pulmonologist would like to start tacrolimus but decided to hold off until the Johns Hopkins evaluation. Pulmonology, nephrology, and rheumatology are currently coordinating care to improve this patient’s disease and quality of life and prolong survival.

Her most recent blood work performed in July 2024 revealed elevated aldolase (11.1 U/L; upper limit 8.1 U/L), which was previously always normal. CPK is now elevated (1394 U/L; normal range 29-143 U/L). She feels great with no clinical signs or symptoms of any active synovitis; on the other hand, muscle weakness is still present but has improved greatly compared to the patient’s first visit to our clinic. 

## Discussion

The diagnostic difficulty in this case is evident. Over the course of five years, multiple specialists have seen our patient, but they have struggled to pinpoint her diagnosis. The news that she has an overlap syndrome with SLE was gut-wrenching to our patient, but she was satisfied that she was finally heard and properly diagnosed. Her rheumatologists considered a diagnosis of fibromyalgia. Such a diagnosis would’ve been detrimental to this patient’s case. There has been a substantial increase in the diagnosis of fibromyalgia, a diagnosis of exclusion, which could potentially increase physician bias in not pursuing further diagnostic testing [3].

It is vital to recognize the signs and symptoms that present with overlap syndromes. Our patient was managed and treated for dermatomyositis for four years prior to her diagnosis with SLE. According to the Systemic Lupus International Collaborating Clinic (SLICC) criteria, our patient fits five criteria, two immunological and three clinical [2]. There have been studies stating overlap syndromes reveal themselves over time and have specific triggers, so she might or might not have had SLE at the time of her DM diagnosis [4,5]. Nonetheless, her diagnosis came after multiple positive biopsies. Her skin biopsy was performed to rule out psoriasis. The findings in her skin biopsy were equivocal, revealing a multitude of differentials including dermatitis herpetiformis, bullous systemic lupus erythematosus, and linear IgA dermatosis. The microscopic description revealed focal parakeratosis and mild spongiosis, perivascular lymphocytic infiltrate, and neutrophilic infiltration of dermal papillae, which are in concordance with the differential diagnoses [6].

Furthermore, dermatomyositis-associated ILD with a positive MDA-5 antibody was linked to a poor prognosis and a rapid progression [7]. In the case report cited, the patient died due to complications despite being treated with methylprednisolone, cyclosporine, cyclophosphamide, and IVIG. The difference is the patient’s age, our patient is much younger than the patient cited, who was 71 at the time of death. Her kidney biopsy wasn’t equivocal. It was a clear-cut lupus nephritis case, with immunofluorescence findings of strong diffuse granular capillary loop reaction to IgG, a mild reaction to C3, and negative for antibodies to phospholipase A2 receptor (PLA-2-R) [8]. Electron microscopy revealed diffuse effacement of podocyte processes and thickened capillary loops with the presence of frequent subepithelial/intramembranous electron-dense deposits. The pathologist’s interpretation was diffuse subepithelial immune complex depositions, which aligns with classic lupus nephritis findings [8].

The management of this patient was and still is difficult. She is resistant to multiple therapies, methotrexate, azathioprine, and mycophenolate mofetil due to side effects. Therapies that relieved her symptoms the most were prednisone, IVIG, and rituximab infusions. Repository corticotropin injections twice weekly were not tolerated; thus, it was reduced to once weekly with questionable improvement in her symptoms. Her pulmonologist and nephrologist are coordinating care regarding the initiation of tacrolimus after her myositis center evaluation. Overlap syndromes are not widely mentioned in the literature, which poses another problem regarding the correct management of these patients. The literature available on overlap syndromes has different combinations of overlapping diseases, rendering its utility variable and potentially limited in certain contexts. It is vital, however, to recognize the importance of diagnosing these cases and how they present. In our case, it took four years for the patient to have her second diagnosis of SLE. While some might argue that this patient with positive anti-RNP antibodies should be considered for a mixed connective tissue disease (MCTD) diagnosis, her severe presentation of lupus (nephritis) and dermatomyositis (ILD) negates that differential [9].

## Conclusions

This case report aims to raise awareness of the complexity and diverse presentations of rheumatologic overlap syndromes. As the prevalence of overlap syndromes increases, it is becoming increasingly important to recognize their unique challenges. Traditional approaches to treating and managing distinct clinical entities may not be sufficient or effective in these cases. The literature suggests that various triggers may contribute to the development of secondary, tertiary, or even quaternary overlap diseases. However, larger studies are necessary to identify these triggers in depth. Rheumatology remains a field with many unknowns, but in this era of accessible knowledge, we have the potential to make significant advancements. By shedding light on overlap syndromes, we can improve the diagnosis, treatment, and overall care of affected individuals.

## References

[1] Iaccarino L, Ghirardello A, Bettio S, Zen M, Gatto M, Punzi L, Doria A (2014). The clinical features, diagnosis and classification of dermatomyositis. J Autoimmun.

[2] Fava A, Petri M (2019). Systemic lupus erythematosus: diagnosis and clinical management. J Autoimmun.

[3] Srinivasan S, Maloney E, Wright B (2019). The problematic nature of fibromyalgia diagnosis in the community. ACR Open Rheumatol.

[4] Pandya R, Lim D, Kleitsch J, Werth VP (2023). Overlap of dermatomyositis and cutaneous lupus erythematosus: a case series. JAAD Case Rep.

[5] Shahidi Dadras M, Rakhshan A, Ahmadzadeh A, Hosseini SA, Diab R, Safari Giv T, Abdollahimajd F (2021). Dermatomyositis-lupus overlap syndrome complicated with cardiomyopathy after SARS-CoV-2 infection: a new potential trigger for musculoskeletal autoimmune disease development. Clin Case Rep.

[6] de Risi-Pugliese T, Cohen Aubart F, Haroche J (2018). Clinical, histological, immunological presentations and outcomes of bullous systemic lupus erythematosus: 10 New cases and a literature review of 118 cases. Semin Arthritis Rheum.

[7] Wu HM, Liu XH, Deng LP (2023). Anti-MDA5 antibody dermatomyositis-associated rapidly progressive interstitial lung disease patient complicated with mixed connective tissue disease: a case report. Int J Rheum Dis.

[8] Garcia-Vives E, Solé C, Moliné T (2019). Antibodies to M-type phospholipase A2 receptor (PLA(2)R) in membranous lupus nephritis. Lupus.

[9] Greidinger EL (2021). Chapter 50 - Overlap syndromes. Systemic Lupus Erythematosus (Second Edition).

